# Bounded risk disposition explains Turing patterns and tipping points during spatial contagions

**DOI:** 10.1098/rsos.240457

**Published:** 2024-10-02

**Authors:** C. M. Jamerlan, M. Prokopenko

**Affiliations:** ^1^ Centre for Complex Systems, Faculty of Engineering, The University of Sydney, Sydney, New South Wales, Australia; ^2^ Sydney Institute for Infectious Diseases, The University of Sydney, Sydney, New South Wales, Australia

**Keywords:** pattern formation, epidemics, opinion polarization, social myths, socio-economic turbulence, susceptibility acquisition

## Abstract

Spatial contagions, such as pandemics, opinion polarization, infodemics and civil unrest, exhibit non-trivial spatio-temporal patterns and dynamics driven by complex human behaviours and population mobility. Here, we propose a concise generic framework to model different contagion types within a suitably defined contagion vulnerability space. This space comprises risk disposition, considered in terms of bounded risk aversion and adaptive responsiveness and a generalized susceptibility acquisition. We show that resultant geospatial contagion configurations follow intricate Turing patterns observed in reaction–diffusion systems. Pattern formation is shown to be highly sensitive to changes in underlying vulnerability parameters. The identified critical regimes (tipping points) imply that slight changes in susceptibility acquisition and perceived local risks can significantly alter the population flow and resultant contagion patterns. We examine several case studies using Australian datasets (COVID-19 pandemic; crime incidence; conflict exposure during COVID-19 protests; real estate businesses and residential building approvals) and demonstrate that these spatial contagions generated Turing patterns in accordance with the proposed model.

## 1. Introduction

Many dynamic processes propagating across space, such as disease transmission, spread of ideas, opinion polarization within a society and social unrest, can be seen as examples of spatial contagion [1]. Several general models have been proposed in the past to characterize and predict contagion dynamics across different domains. For instance, the spatial spread of infectious diseases, diffusion of rumours and emergence of consensus were modelled in a unified setting by Balcan and Vespignani [2]. An epidemiological model for riot participation was developed by Davies *et al*. [3] to examine emergent patterns of large-scale social disorder and violence. Different social dynamics, including Schelling-style segregation, spreading of social myths and rioting behaviours were also considered as instances of a generic susceptible-infected-susceptible (SIS) model [4]. A model proposed by Braha [5] explored the similarity between social instability, unfolding natural hazards and epidemics.

Three contagion categories (disease, addiction and rumour) were considered with respect to behavioural characteristics, including imitative behaviour and intentionality [6]. A common feature of these approaches is a representation of spatial connectivity through either a network of locations or a geographical proximity matrix (e.g. distances in a spatial interaction model), complemented by a description of (population) mobility.

Yet, concise generic modelling of spatial contagions and prediction of collective dynamics remains a challenge [7–9]. Importantly, in addition to multiple contagion types, there is a variety of human behaviours shaped by individual risk perception and risk disposition factors that affect the collective contagion dynamics. A canonical threshold model of collective dynamics, developed by Granovetter [10], examined individual thresholds for joining a ‘behavioural contagion’, such as riot, strike or information diffusion, with thresholds defined as the proportion of already-engaged participants. One of the key insights of the threshold model was that ‘groups with similar average preferences may generate very different results’ [10]. The association between risk perception and behaviour was also found to be unstable in more recent studies of the influenza pandemic [11]. However, during the COVID-19 pandemic, risk perception was found to significantly correlate with the reported adoption of preventive health measures across very different cultural backgrounds [12].

Risk aversion and unanticipated effectiveness were identified as two salient factors producing extreme market shares in another canonical information contagion model [13]. Complex civil disorder models with heterogeneous reactive agents (e.g. active and inactive rioters) included additional parameters beyond risk aversion, such as perceived hardship, political grievance and threshold for rebelling [14]. In addition, a fear of social isolation was proposed as an inherent key factor in generating behavioural contagions in public opinion by Krassa [15], following the ‘spiral of silence’ theory [16]. Crucially, the majority of risk perception and disposition elements may vary along a spectrum, thus allowing for continuous variations rather than adhering to rigid binary distinctions. In general, a combination of these elements could shape the collective vulnerability of the population to a specific contagion.

Another factor complicating the modelling of spatial contagion is population mobility [17]. This has been considered in the context of epidemics, natural disasters, opinion polarization or conflict situations, as individuals strive to navigate contagion or social dynamics while seeking safety [18,19], (dis-)engaging with different groups [3,20,21] or following social myths and beliefs [22,23]. Importantly, there is a feedback loop between the unfolding epidemic/social dynamics and population mobility. A canonical example is population segregation, occurring when neighbourhoods homogenize due to social, economic or racial factors, leading to further segregation as individuals continue to express preference towards their own group [20]. In general, movement or resettlement of individuals who respond to (perceived) risks and benefits produces intricate geospatial patterns, which may represent either population segregation or opinion/belief distribution [4,24–26].

Thus, our study is motivated by the need to develop a unifying framework modelling several contagion dynamics, ranging from epidemics to opinion dynamics, civil unrest and boom-and-bust investments. Ideally, such a framework should represent risk-driven decision-making and population mobility, and be capable of identifying tipping points in contagion dynamics, enabling rigorous analysis of socio-economic and socio-political ramifications.

Our primary objective is the characterization of pattern formation across different types of spatial contagion and underlying human risk disposition. Canonical epidemiological and information propagation models, such as SIS [27], susceptible-infected-recovered (SIR) [2,28] and susceptible-infected-recovered-susceptible (SIRS) [29], typically consider homogeneous populations and do not differentiate across risk disposition elements. Several recent studies attempted to enhance epidemic/social dynamics models with representations of behaviours using varying degrees of rationality towards risks [4,30,31]. However, the effects of varying individual susceptibility to infection on the spatial distribution of the contagion remain underexplored.

In epidemic modelling, waning immunity or acquired susceptibility within a population plays a pivotal role in characterizing infection propagation [32–34]. In general, susceptibility acquisition may influence other types of spatial contagion. For example, Davies *et al.* [3] expressed the rioting phenomenon as an epidemic-like process with susceptibility, where ‘susceptible’ individuals transition to an ‘infected’ rioting state once the exposure to ambient disorder incites participation. Yet, while previous studies considered susceptibility within social unrest models [3] or information propagation models [35,36], they did not explicitly model susceptibility acquisition, staying within the scope of SIR modelling. On the contrary, Harding *et al.* [4] considered the SIS model with recovered individuals losing immunity completely, but did not investigate the range of partial susceptibility acquisition.

Thus, our second objective is to explicitly examine susceptibility acquisition as a general process applicable not only to epidemics, but also to broad social dynamics. In doing so, we will focus on the role of susceptibility acquisition in pattern formation during spatial contagion. Crucially, by considering the generalized susceptibility acquisition as a variable parameter, which can smoothly transition along a spectrum (from permanent immunity in SIR dynamics, to complete loss of immunity in SIS dynamics), we can identify critical regimes, i.e. phase transitions or tipping points, which mark substantial shifts in contagion dynamics across several societal contexts. This will significantly enhance previous studies of criticality in spatial contagions [2,4,31,35,37] by providing a more generic, unified view on the phase transition mechanisms and the resultant contagion patterns.

In summary, we aim to (i) map individual risk disposition to patterns formed during spatial contagion within a concise unifying framework, (ii) informed by this framework, investigate risk disposition elements within their bounds and identify critical points, and (iii) examine the generalized susceptibility acquisition as a factor driving pattern formation and tipping points.

To achieve these objectives, we propose a concise conceptual framework comprising different types of contagion within a coherent vulnerability space comprising risk disposition elements. Using a generalized SIRS epidemiological model and the maximum entropy principle, we model mobility flows that change dynamically in response to the current contagion state and the geospatial population distribution. We then investigate the resulting spatial patterns and critical regimes emerging within the vulnerability space.

In addition to the transitions across well-known spatial patterns reminiscent of Turing patterns in reaction–diffusion systems (e.g. spots → labyrinth → gaps) [38–42], or risk disposition parameters (similar to loss of tolerance [20]), we identify novel transitions in the space of generic susceptibility acquisition. To validate the model, we investigate the progression of the COVID-19 pandemic, crime incidence, conflict exposure during COVID-19 protests, real estate businesses and residential building approvals in Australia, specifically, in the most populous state of New South Wales (NSW). We show that the resultant configurations of these geospatial contagions exhibit Turing patterns with percolation parameters falling within the range identified by the model.

## 2. Results

### 2.1. Conceptual framework

Risk disposition typically refers to an individual’s overall attitude or approach towards risk, comprising elements of risk aversion, risk-taking behaviour, risk tolerance, risk acceptance, risk mitigation strategies and so on [43–46]. In general, risk disposition is influenced by various factors, including psychological and emotional states, past experiences, cultural influences and situational context. In this study, we limit our analysis to just a few factors, exemplifying a larger and more generic space. We shall refer to this high-dimensional space as the *contagion vulnerability* space, within which we characterize the overall vulnerability of the affected populations to the contagion, given the risk disposition of the individuals. In other words, each combination of risk disposition features in this space dynamically generates a specific spatial contagion pattern spanning across locations and affecting the population.

**Figure 1.**
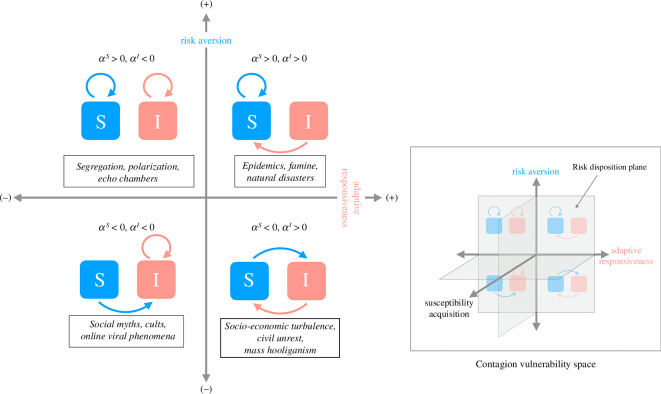
The risk disposition plane and the contagion vulnerability space. Each spatial contagion type (with examples boxed) is characterized by a specific risk disposition combination in terms of adaptive responsiveness (x-axis) and risk aversion (y-axis). Adaptive responsiveness grows from left to right (i.e. as the x value increases), and risk aversion grows from bottom to top (i.e. as the y value increases). Modelling these risk disposition combinations (counter-clockwise from top right) produces: an epidemic-like contagion where both affected (*I*) and susceptible (*S*) individuals seek to survive and escape the contagion when risk aversion and adaptive responsiveness are both positive (top right); Schelling-style segregation where positive risk aversion and negative adaptive responsiveness drives people to stay in like-minded groups (top left); social myths or online viral trends where people seek and flock to groups ‘affected’ by the information contagion due to negative risk aversion and adaptive responsiveness (bottom left); and war or civil unrest where negative risk aversion and positive adaptive responsiveness compels individuals to seek opposite groups (bottom right). Different types of contagion dynamics map to four combinations of bounded risk disposition parameters (adaptive responsiveness $\alpha^{I}$ and risk aversion $\alpha^{S}$), with quadrants defined by varying the signs of the corresponding Lagrange multipliers $\alpha^{I}$ and $\alpha^{S}$ (see §2.2).

In order to map risk disposition and various contagion dynamics, we begin with two dimensions, which form a plane in a higher dimensional *contagion vulnerability* space, as illustrated in figure 1.

The vertical dimension, labelled ‘risk aversion’, characterizes the individual’s general reluctance to take risks, leveraging their capacity to anticipate future risks and take preventive action. Risk aversion may be partial or bounded, and hence this notion is distinct from ‘risk avoidance’, which is a strategy aimed at entirely sidestepping or eliminating exposure to particular risks. For example, during a pandemic, a healthy but susceptible individual may anticipate the risk of infection in locations where the contagion is highly prevalent, thus motivating the choice to minimize exposure to such locations.

The horizontal dimension characterizes the individual’s tendency to adapt their response to an ongoing disruption or upheaval, such as pandemic, natural disaster, war, socio-economic crisis, etc. We label this dimension as ‘adaptive responsiveness’ to reflect that it quantifies the adaptability needed to generate a response to present circumstances, rather than the capacity to anticipate and reduce future risks. For instance, highly adaptive individuals may respond to disruptive situations by seeking safer locations once they encounter and experience the disruption.

In this study, we do not aim to identify complex underlying mechanisms and biases that shape individual risk disposition, due to psychological, cognitive, social and cultural factors. Instead, we consider the boundedness of risk aversion and adaptive responsiveness in a mechanism-independent way. Our assumption is that it is possible to arrange values of bounded aversion and responsiveness on their respective axes using linear order. In other words, it is possible to establish a consistent directionality, relating the points to each other on the basis of their position along the axis, and thus, allowing for the determination of their relative positions.

We also assume that the values of both risk aversion and adaptive responsiveness can be positive or negative. While a positive risk aversion indicates the preference to reduce threats and risks in general, a negative risk aversion represents the opposite tendency to seek risky situations, i.e. risk-taking behaviour. As an aside, we note that positive or negative risk aversion should not be interpreted as avoidance of ‘positive’ or ‘negative’ risks [47]. In our interpretation, we assign the positive or negative direction to the ‘aversion’ tendency, rather than attribute positive or negative quality to the nature of risk itself. Similarly, a positive adaptive responsiveness captures the extent of adaptability in escaping dangerous or unconventional situations, while a negative adaptive responsiveness specifies the entrenched preference to continue experiencing such circumstances.

Finally, we assume that these dimensions are independent of each other, quantifying the capacities to either assess *future* risks or respond to *ongoing* crises, upheavals or transformations. Importantly, we argue that the quadrants formed on the risk disposition plane of the contagion vulnerability space correspond to distinct contagion types.

The top-right quadrant (figure 1) represents contagion scenarios typical for epidemics and natural disasters. In these scenarios, we may observe positive risk aversion, so that susceptible individuals become repelled by risks and prioritize their personal survival by striving to stay unaffected. Concurrently, the affected (infected) individuals show positive adaptive responsiveness, i.e. the tendency to respond to surrounding present dangers, thus trying to escape the contagion [4].

The top-left quadrant of the risk disposition place describes the combination of negative adaptive responsiveness (when individuals affected by some ongoing disruption prefer to keep the ‘status quo’), and positive risk aversion (when some impending threats compel unaffected individuals to reduce their exposure to risks), see figure 1. This combination may induce population segregation, where individuals tend to gravitate towards like-minded groups, thus reinforcing their pre-existing beliefs during a cultural upheaval or paradigm shift [35]. While this is analogous to population segregation [20], we note that in Schelling’s model of segregation, individuals would relocate and resettle in different geographical locations, while in our model, we will consider the temporary mixing of individuals without permanent resettlement. Opinion polarization, as well as the formation of echo chambers in social media platforms, exemplify other dynamics resulting from this combination, when a personal prejudice and lack of tolerance towards individuals with perceived dissimilarity hinder constructive interactions [35,48].

Considering the bottom-left quadrant (figure 1), we note that negative adaptive responsiveness is also apparent in scenarios leading to the emergence of social myths, cult formation and the spread of online viral phenomena. In these instances, affected individuals continue to engage with the unfolding contagion, which fosters a widespread propagation of (mis-)information or beliefs. At the same time, a negative risk aversion among unaffected but susceptible individuals drives them to associate with affected individuals and adopt their potentially risky beliefs and practices [23,49].

Finally, the bottom-right quadrant (figure 1) illustrates the scenarios, which can be observed during periods of conflict, civil unrest or socio-economic turbulence, e.g. ‘boom-and-bust’ investment cycles. For example, a rioting situation or an asset price bubble may incite disorder participation or investment activity, reinforced by negative risk aversion (i.e. risk taking), so that ‘susceptible’ individuals may seek to become ‘infected’ en masse [3,50,51]. At the same time, positive adaptive responsiveness of the ‘infected’ individuals ensures that the important mode of self-preservation induces the affected people to flee or take profit, and thus recover from the contagion.

Importantly, when comparing the top and bottom parts of the plane, we note that at the top, susceptible individuals tend to reduce their exposure to the contagion, while at the bottom, they may seek to join the affected, ‘infected’, state. Contrasting the left- and right-hand sides of the plane, we point out that negative adaptive responsiveness tends to keep affected individuals accepting the contagion, while positive adaptive responsiveness manifests itself in the tendency of affected individuals to evade, rather than accept, the contagion.

This conceptual framework maps various risk disposition attitudes to the corresponding contagion dynamics that they produce. The considered two-dimensional space allows us to investigate bounded risk aversion and adaptive responsiveness with respect to contagion dynamics. Once the model is parametrized, these parameters and their ranges can be formally defined. This two-dimensional space is not meant to be exhaustive and can be extended by incorporating other dimensions. In fact, a third dimension is formed by the generalized susceptibility acquisition, and one can vary this parameter for every negative and positive risk disposition combination. For example, in the top-right quadrant, it pertains to increased susceptibility to disease during epidemics. In the top-left quadrant (segregation), susceptibility acquisition represents an increased prejudice, i.e. reduced tolerance towards outsiders. In the bottom-left quadrant, it captures an increased susceptibility to believing social myths. Finally, in the bottom-right quadrant (war and civil unrest), susceptibility acquisition describes a higher tendency to participate in social disorder.

In general, this conceptual framework can be extended to represent multiple independent, orthogonal, risk disposition dimensions, with the aim to associate the resultant contagion types with orthants (hyperoctants) in an n-dimensional contagion vulnerability space. Some variables, for example, susceptibility acquisition, could range only between 0 and ∞, limiting the number of generated contagion (sub-)types.

### 2.2. Mapping contagion types to risk disposition combinations

The canonical SIRS epidemic model groups individuals into three compartments: susceptible (S), infected (I) and recovered (R) [32]. The compartmental dynamics depends on transmission rate β, recovery rate γ and the loss of immunity ζ, as described in Methods (§4.1). Loss of immunity or, essentially, acquisition of susceptibility to contagion, can be generalized from the epidemic SIRS context to other contagion types considered above (i.e. the risk disposition attitude combinations). In doing so, we introduce the generalized susceptibility acquisition as an abstract variable ζ (originally defined as susceptibility to infection). In the case of opinion polarization or Schelling-style segregation, ζ represents the prejudice of individuals against those outside of their own group, with a higher ζ catalysing opinion polarization and the formation of echo chambers. Susceptibility acquisition in the context of social myth spreading, cult formation and online viral phenomena takes the form of the tendency to accept or adopt widespread beliefs or actions, with higher ζ reflecting an increased tendency. Finally, susceptibility acquisition during conflicts, civil unrest or socio-economic turbulence represents the inclination to engage in disruptive behaviours like rioting, violence, or mass hooliganism or in asset price bubbles. A higher ζ for this case indicates a growing inclination towards civil disobedience or increasing fear of missing out on some investment opportunity.

In our study, we adopted the Boltzmann–Lotka–Volterra (BLV) methodology [52], which models a broad range of socio-economic dynamics by combining the principle of maximum entropy with the Lotka–Volterra model of population dynamics [4,53–55]. The BLV approach has two components: a slow dynamic, which, in our model, characterizes the progression of the contagion (modelled by SIRS), and a fast dynamic, determining population mobility flows, which are dependent on the contagion state. Following [4], these mobility flows are characterized by the movement of susceptible and affected (infected) populations, denoted by $\phi_{ij}^{S}$ and $\phi_{ij}^{I}$, respectively.

To account for population mobility, we generalized a multi-city SIR network model originally proposed by [17], extending it to SIRS dynamics and describing the dynamics of disease transmission within a meta-population of individuals who regularly travel between multiple locations. Each location i has a sub-population $P_{i}$, with $I_{i}$ representing affected individuals, $S_{i}$ representing susceptible, and $R_{i}=P_{i}-S_{i}-I_{i}$ representing recovered, and a location benefit $b_{i}$ defined by the local contagion state as $b_{i}=\frac{P_{i}-I_{i}}{P_{i}}$ (i.e. the fraction of unaffected individuals), as described in Methods (§4.2, see equations (4.4)–(4.6)).

The optimal solutions for mobility flows $\phi_{ij}^{S}$ and $\phi_{ij}^{I}$ produced by the BLV model (see equations (4.11) and (4.12)) depend on two Lagrange multipliers $\alpha^{I}$ and $\alpha^{S}$, which correspond to system constraints. Importantly, these Lagrange multipliers serve as bounded risk disposition parameters with respect to perceived benefits driving mobility towards (or away from) a location. In general, however, these Lagrange multipliers could be positive or negative, capturing directionality (attraction or repulsion). Parameter $\alpha^{I}$ quantifies the tendency with which affected (‘infected’) individuals judge the benefit of pursuing ($\alpha^{I}$ < 0) or avoiding ($\alpha^{I}$ > 0) affected locations (interpreted as their response to the contagion, i.e. accepting or fleeing their current situation). Parameter $\alpha^{S}$ quantifies the weight that non-affected (susceptible) individuals put on the benefit of moving to ($\alpha^{S}$ < 0) or avoiding ($\alpha^{S}$ > 0) the affected locations, interpreted as their risk aversion of contagion. While the two Lagrange multipliers $\alpha^{I}$ and $\alpha^{S}$ appear in the model symmetrically, our interpretation captures the asymmetry of benefits $b_{i}$ defined as non-contagion preferences. This emphasizes that changes in $\alpha^{I}$ represent varying levels of acceptance or rejection of current circumstances, while changes in $\alpha^{S}$ indicate the shift of mixing preference towards or away from the affected (‘infected’) groups. By varying the signs and values of the bounded risk disposition parameters ($\alpha^{I}$ and $\alpha^{S}$), one can examine different combinations within the defined risk disposition plane.

As a result, we categorized risk disposition combinations (informally defined in §2.1) into four primary types, mapping directly onto the four quadrants of the risk disposition plane (figure 1). Figure 1 shows that each quadrant was mapped to a unique combination of signs of Lagrange multipliers $\alpha^{I}$ and $\alpha^{S}$. For example, the top-right quadrant represents epidemic or natural disaster dynamics where both susceptible and affected (infected) individuals prefer to evade the contagion: they tend to be attracted to destinations with a higher benefit expressed as a lower fraction of affected individuals. Conversely, the bottom-left quadrant captures ‘infodemic’ dynamics when both susceptible and affected individuals are attracted to destinations with a higher fraction of affected individuals (i.e. lower benefit).

### 2.3. Pattern formation in the contagion vulnerability space

**Figure 2.**
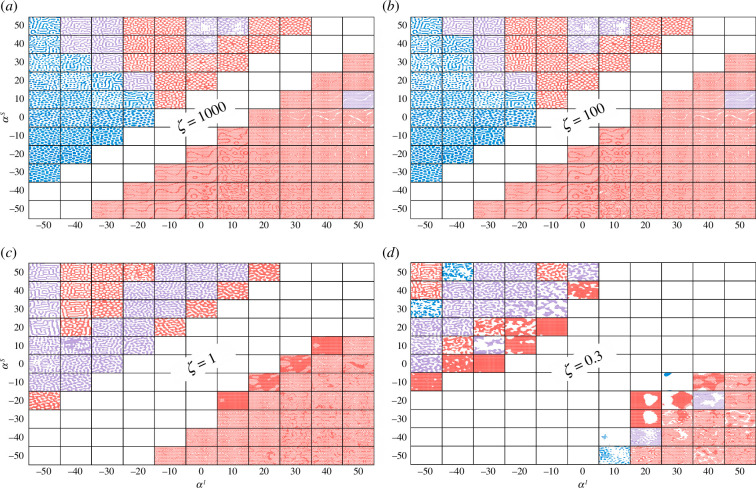
Pattern formation in the space of $\alpha^{I}$ and $\alpha^{S}$ for transmission rate β=10, recovery rate γ=5, and different values of susceptibility acquisition ζ. Each $\alpha^{I}$–$\alpha^{S}$ quadrant is modelled as 11×11 grid of lattice configurations, with each configuration comprising a 40×40 lattice of locations. The configuration colour is chosen according to the average size of connected clusters $\left\langle \hat{r}\right\rangle$ within the configuration (i.e. percolation order parameter), distinguished by the order of percolation magnitude that separates pattern types: white when $\left\langle \hat{r}\right\rangle$ is undefined, blue when $0<\langle \hat{r}\rangle \leq 0.04$, purple when $0.04<\langle \hat{r}\rangle \leq 0.4$, and red when $0.4<\langle \hat{r}\rangle \leq 1$. Configurations with well-formed patterns are clearly separated into distinct phases for higher susceptibility acquisition approaching SIS dynamics, at (*a*) ζ=1000 and (*b*) ζ=100. For a medium ζ, (*c*) ζ=1, emerging patterns can be identified as coexisting within mixed modes. Finally, pattern types are only beginning to emerge at lower susceptibility acquisition, (*d*) ζ=0.3, where the dynamics approach SIR dynamics.

By systematically exploring the space of risk disposition parameters ($\alpha^{I}$ and $\alpha^{S}$) and generic susceptibility acquisition ζ, we identified specific phase dynamics and critical regimes for different contagion types. Figure 2 illustrates the resultant patterns across the contagion vulnerability space for several important values of susceptibility acquisition ζ, with the infection levels in each location binarized relative to the mean infection level across the lattice.

We observed five distinct pattern types in the $\alpha^{I}$–$\alpha^{S}$ phase space: (i) the ‘uniform’ pattern: a uniform spatial distribution of infection states along the middle diagonal (shown in white), (ii) the ‘spots’ pattern: isolated clusters of infection (shown in blue), (iii) the ‘labyrinth’ pattern: highly connected maze-like regions (shown in purple in the top half), (iv) the ‘gaps’ pattern: interconnected web-like regions with scattered empty areas (shown in red in the top half), and (v) the ‘chequerboard’ pattern: mixed-mode regions with a chequerboard structure (shown in red and purple in the bottom half).

**Figure 3.**
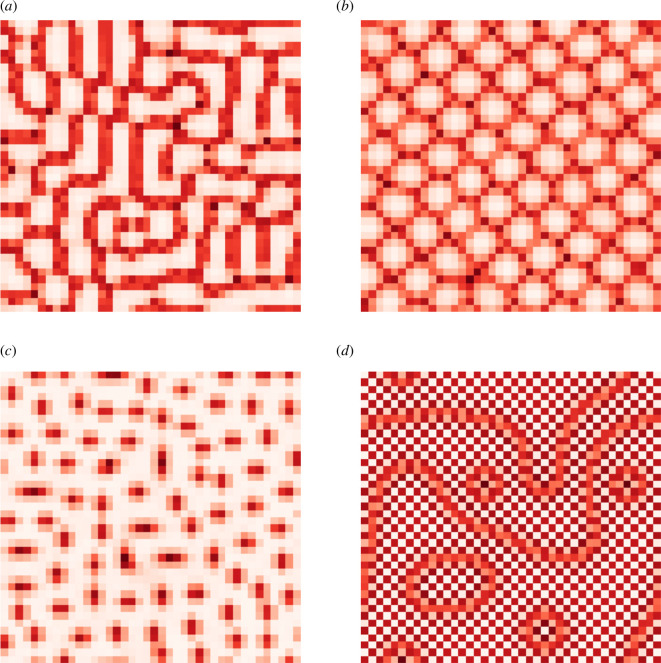
Types of spatial contagion patterns: (*a*) labyrinth, observed in polarization dynamics, (*b*) gaps, observed in polarization dynamics and epidemics, (*c*) spots, observed in polarization dynamics and social myths spreading, and (*d*) chequerboard with overlaid strands and rings, observed mostly in socio-economic turbulence dynamics and social myths spreading.

The observed spots, labyrinth and gaps patterns (shown in more detail in figure 3*a–c*) are directly comparable to well-known Turing patterns, which emerge in reaction–diffusion systems: hexagons (spots), stripes (labyrinthine patterns), or reverse hexagons (honeycombs or gaps) [38,39]. Previously, the chequerboard pattern below the uniform diagonal was characterized as an anti-aligned phase of SIS dynamics—this was carried out by computing the staggered magnetization parameter [4], see §4. In our generalized SIRS study, the mixed-mode phase, observed below the diagonal (coloured in red), with strands and rings emerging on top of the underlying chequerboard background (shown in figure 3*d*) may represent two disparate scales of interaction on a ‘superlattice’: fine-grained (chequerboard) and course-grained (strands and rings).

### 2.4. Critical regimes in the contagion vulnerability space

To characterize critical regimes, we quantified notable shifts between the resultant pattern types (phases) using methods of percolation theory, as described in §4.3.

Crucially, we observed that configurations with different patterns are close to each other in the $\alpha^{I}$–$\alpha^{S}$ phase space. In other words, the well-defined phases are separated by transitions across pattern types (e.g. spots → labyrinth → gaps), driven by changes in the bounded risk disposition parameters. The placement of configurations with the labyrinth pattern between spots and gaps is in concordance with previous studies where labyrinthine patterns were typically observed when changing an underlying control parameter along a protocol: initially producing hexagons (spots) and then generating reverse hexagons (gaps) [38–40].

The boundaries of the observed phases do not coincide with the four quadrants forming the risk disposition plane, and different spatial patterns may thus characterize the same contagion type. For example, the top right quadrant (epidemics) includes both gaps and chequerboard patterns. This diversity implies that although both susceptible and affected individuals share the same (positive) bounded risk disposition subspace, the relative differences in the extents of bounded risk disposition (e.g. $\alpha^{S}>\alpha^{I}$) produce distinct spatial patterns (e.g. gaps). Similarly, three pattern types (gaps, labyrinth and spots) are observed for opinion polarization, illustrated in the top-left quadrant, given variations in bounded risk disposition: $\alpha^{S}<-\alpha^{I}$ for spots, and $\alpha^{S}>-\alpha^{I}$ for labyrinth and gaps. In general, the presence of distinct phases strongly indicates that the dynamics are separated by phase transitions during which the system undergoes significant reconfigurations triggered by small changes in system parameters.

Interestingly, when one of the risk disposition parameters is close to zero (indifference) while the other is near maximal (almost perfect risk aversion or full adaptive responsiveness), the patterns become more irregular. This is illustrated by ‘incursions’ of malformed labyrinth-like patterns among gaps and chequerboard patterns in the top-right quadrant (SIRS epidemic dynamic).

For small values of susceptibility acquisition (ζ≤1), such mixed regimes are even more prominent (figure 2*c,d*). The reason is that for small ζ, SIRS dynamics degenerate into SIR-like dynamics, which for the pure SIR case, should result in zero infections.

### 2.5. Tipping point in susceptibility acquisition

Importantly, we identified a tipping point (phase transition) in the space of susceptibility acquisition ζ. The dynamics is at the sub-critical phase at ζ=0.3 (figure 2*d*), with patterns only beginning to emerge. For example, the top-left quadrant (opinion polarization) shows mostly labyrinth patterns, which are yet to separate into spots and gaps, while the bottom-right quadrant (socio-economic turbulence) shows a mix of highly irregular patterns. Furthermore, the patterns corresponding to epidemic and social myth contagions have not yet formed (top-right and bottom-left quadrants).

As ζ is increased to 1 (figure 2*c*), well-defined phases with clear patterns start to emerge (e.g. for epidemics and social myth spreading) or even stabilize (e.g. for polarization and socio-economic turbulence). At ζ=100 and ζ=1000, the dynamics settle into distinct patterns separated by phase transitions in space of the bounded risk disposition parameters. At higher ζ values (ζ>10), we recovered resultant patterns of SIS dynamics [4].

**Figure 4.**
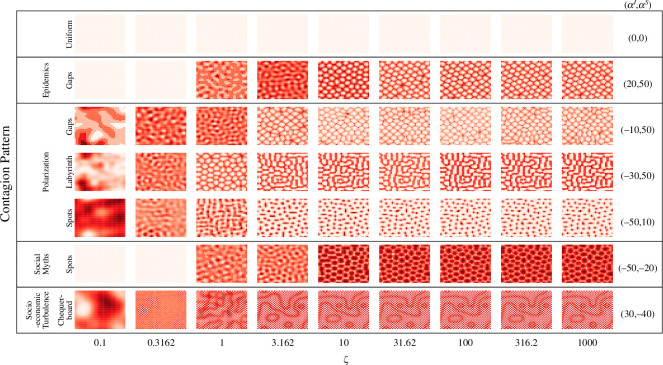
The development of patterns (uniform, labyrinth, chequerboard, spots and gaps) in the space of susceptibility acquisition ζ. Representative configurations for each pattern were selected from figure 2, and their $\alpha^{I}$–$\alpha^{S}$ coordinates are indicated in the right-most column. The ‘infection’ values were normalized for each configuration determined by $\alpha^{I}$, $\alpha^{S}$ and ζ. Locations with higher relative ‘infection’ levels are shown with darker colours.

To investigate the role of susceptibility acquisition in pattern formation for each contagion type, we quantified and traced the transitions with respect to changes in ζ for selected configurations. These configurations represent different spatial contagion types. Figure 4 illustrates the development of each pattern type from ζ=0.1 to ζ=1000 on a logarithmic scale. Across all contagion types, except socio-economic turbulence, a labyrinth-like proto-pattern is observed for small values of ζ in all patterns. This precedes the emergence of eventual gaps, labyrinth and spots. For epidemics and social myth spreading, this proto-pattern emerges at ζ≈1, while for polarization, it emerges for a slightly smaller susceptibility acquisition, ζ≈0.3. As ζ increases, the proto-pattern differentiates and develops into corresponding stable forms becoming ‘gaps’ (for epidemics) and ‘spots’ (for social myths), or ‘spots’, ‘labyrinth’ and ‘gaps’ (for polarization). Social unrest dynamics has its own proto-pattern emerging between ζ≈0.3 and ζ≈1 before becoming a mixed-mode ‘chequerboard’ overlaid with strands and rings. These patterns remain unchanged as the dynamics approach SIS form with higher ζ.

**Figure 5.**
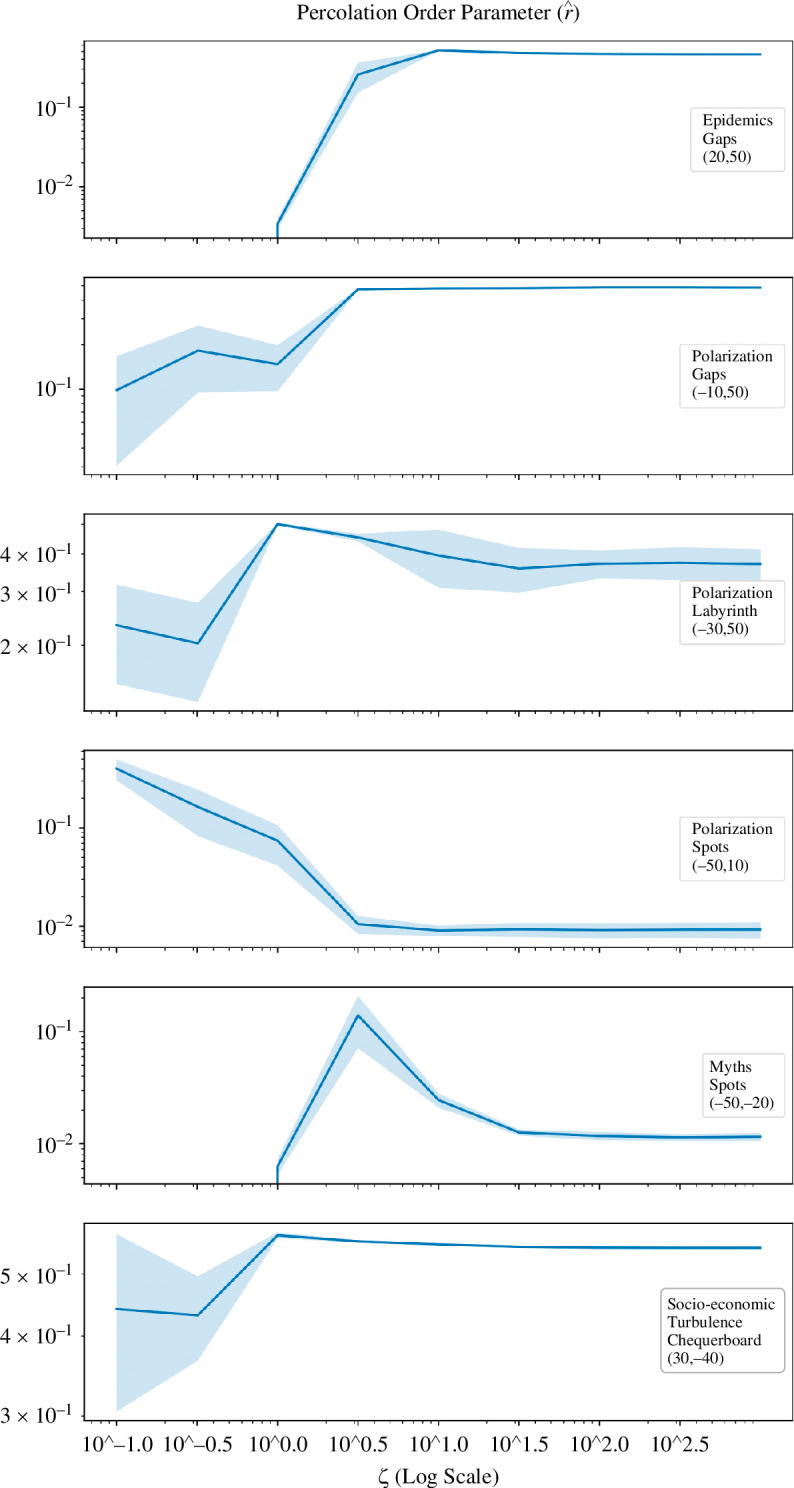
Changes in the average size of connected clusters $\left\langle \hat{r}\right\rangle$, plotted against the susceptibility acquisition ζ, with both axes on a logarithmic scale, for the representative configurations shown in figure 4. Shaded areas show the standard deviation over 10 runs for a given configuration determined by $\alpha^{I}$, $\alpha^{S}$ and ζ. The plot for the uniform contagion patterns where $\left\langle \hat{r}\right\rangle$ is undefined due to absence of clusters is not shown.

For different pattern types, the critical regime indicated by a notable change in the average size of connected clusters $\left\langle \hat{r}\right\rangle$, with respect to changes in ζ, was observed at slightly different critical values $\zeta_{c}$. For example, in polarization dynamics, the critical point for all three pattern types (labyrinth, spots and gaps) is observed in the range $0.3<\zeta_{c}<3$ (see figure 5). The socio-economic turbulence dynamics (chequerboard pattern) also exhibits criticality in this range. On the other hand, for epidemic dynamics (the gaps patterns) and social myth spreading (the spots patterns) a critical change was observed in the range $1<\zeta_{c}<10$, as shown by increases in the percolation order parameter (see figure 5). Inspecting equation (4.3) reveals that, in proximity to critical regime, susceptibility acquisition in the range 1<ζ<10 can balance, to some degree, the recovery dynamics shaped by the recovery rate γ=5.

In summary, there is a change in patterns observed in response to changes in all three considered parameters ($\alpha^{I}$, $\alpha^{S}$ and ζ) and these changes can be identified with phase transitions in the corresponding parameter space.

### 2.6. Case study: geospatial spread of COVID-19 in New South Wales, Australia

We examined the COVID-19 pandemic spread in Australia within the most populous state of NSW, and analysed the geospatial distribution of the COVID-19 daily incidence reported by NSW Health authorities [56]. Specifically, we focused on three key dates during the pandemic stage shaped by the B.1.617.2 (Delta) and the B.1.1.529 (Omicron) variants of SARS-CoV-2 in 2021: (i) 4 September, when the first peak, driven by the Delta variant, was registered in NSW (1507 daily COVID-19 cases, i.e. approximately 185 daily cases per million); (ii) 21 December, when the Omicron variant started to dominate the Delta variant (3264 daily COVID-19 cases, i.e. about 400 daily cases per million); and (iii) the next peak, on 31 December, created by the rapid spread of the Omicron variant in NSW (22 201 daily COVID-19 cases, or over 2740 daily cases per million).

**Figure 6.**
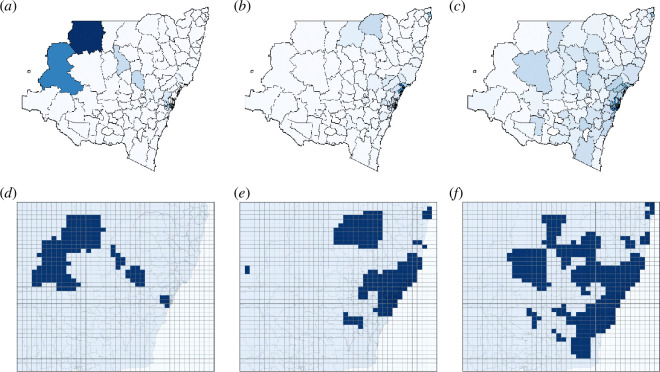
COVID-19 incidence levels in NSW, Australia are shown across three key dates during the period of peak infection. (*a*)*–*(*c*) Shows areas separated by LGA boundaries. Darker regions illustrate higher levels of relative infection. (*d*)*–*(*f)* Shows the redistributed incidence data on the overlaid grid, where areas with incidence fractions (number of infected over the population) exceeding the mean incidence fraction across the grid are indicated in blue. The normalized average cluster sizes $\left\langle \hat{r}\right\rangle$ for each configuration are shown in table 1.

For each considered date, we produced a choropleth map of the normalized incidence across local government areas (LGAs) in NSW, visualized in figure 6. Then, we discretized each choropleth map into a 40×40 grid, comparable with the lattice configurations used in the pattern formation and percolation analysis for the generalized SIRS network (multi-city) model. Finally, we binarized the renormalized incidence in each grid cell relative to the mean across the grid, producing contagion patterns. More details of this process are provided in §4.4 and the electronic supplementary material.

**Table 1.** Average cluster size (percolation order parameters) for COVID-19 incidence in NSW.

	4 September 2021	21 December 2021	31 December 2021
	(first peak, Delta variant)	(inter-peak)	(second peak, Omicron variant)
$\left\langle \hat{r}\right\rangle$	0.07251	0.04795	0.1817
normalized $\left\langle \hat{r}\right\rangle$	0.1237	0.08179	0.3100

Figure 6*a–c* shows the NSW choropleth maps with LGA boundaries. Figure 6*d*–*f* shows the discretized and renormalized incidence data on the 40×40 grid, highlighting the areas with incidence fraction (the number of infected individuals within a grid cell relative to the population) exceeding the mean normalized incidence fraction across all cells. The highlighted areas with above-the-mean incidence form a pattern of contagion on the corresponding date, and can be examined in terms of the percolation order parameter, i.e. the average cluster size (ACS) $\left\langle \hat{r}\right\rangle$.

Table 1 shows the resulting ACSs $\left\langle \hat{r}\right\rangle$ for each date, along with the normalized $\left\langle \hat{r}\right\rangle$ obtained with respect to the maximum $\left\langle \hat{r}\right\rangle$ attainable for a fully percolated (infected) grid. We report that these values fall within the thresholds for labyrinthine patterns ($0.04<\langle \hat{r}\rangle \leq 0.4$), decreasing from the first peak (the Delta-induced wave) to the inter-peak point (rapid spread of the Omicron variant), and increasing on the date of the second peak (the Omicron-induced wave).

Comparing these patterns with contagion patterns produced by the generalized SIRS (multi-city) network model, we note that patterns formed in the top-right quadrants of figure 2 (i.e. the patterns corresponding to epidemic dynamics with positive $\alpha^{I}$ and $\alpha^{S}$, across all considered values of ζ) contain only labyrinths and gaps. Specifically, the labyrinthine patterns are concentrated in areas where the healthy but susceptible individuals are highly risk-averse (i.e. avoid locations with elevated infection levels), while the infected individuals have a low or near-zero adaptive responsiveness (i.e. do not seek locations with low infection levels). Arguably, this combination can be expected when pandemic response includes non-pharmaceutical interventions, e.g. lockdowns characterized by partial compliance with ‘stay-at-home’ orders, and the pandemic response in NSW during the examined period indeed employed large-scale lockdown policies [57–61].

### 2.7. Case study: criminal records (incidents of assault) in NSW, Australia

**Figure 7.**
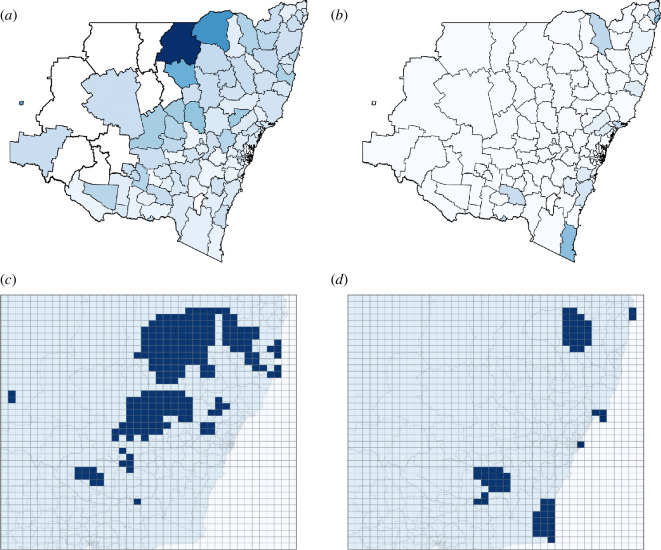
Criminal records (incidents of assault) for 2022–2023 and conflict exposure during protests against the COVID-19 lockdown in July–December 2021 are illustrated for each LGA in NSW, Australia. Top sub-figures show that the darker LGAs have a higher number of (*a*) assault incidents, or (*b*) a higher conflict exposure for COVID-19-lockdown-related protests (normalized to the LGA’s population). Bottom sub-figures show (*c*) the crime incidence and (*d*) the conflict exposure in protests, redistributed on the overlaid grid, where areas with the crime incidence (number of assault events divided by LGA population) or the conflict exposure (estimated affected population divided by LGA population) exceeding the mean value across the grid are indicated in blue. The normalized ACSs $\left\langle \hat{r}\right\rangle$ for each configuration are shown in table 2.

We analysed criminal incidents (assaults) for 2022–2023 in NSW recorded by the NSW Bureau of Crime Statistics and Research [62]. This dataset contains an annual snapshot of assault incidents for each LGA. Figure 7 (left column) shows LGAs with higher relative criminal incidence (normalized by the corresponding LGA population) in darker shades for both the choropleth map (figure 7*a*) and the binarized overlaid grid map (figure 7*c*). Table 2 shows the corresponding ACS $\langle \hat{r}\rangle =0.09656$, which falls within the threshold for labyrinthine patterns ($0.04<\langle \hat{r}\rangle \leq 0.4$). While not definitive, it could be argued that distribution of criminal activity across LGAs may be correlated with some segregation of social and anti-social groups, neighbourhood violence and criminogenic environments [63–65].

**Table 2.** ACS (percolation order parameters) for criminal records (incidents of assault), COVID-19-lockdown-related protests, real estate businesses and building approvals in NSW.

	criminal	COVID−19 protests	real estate businesses and building
	records	(conflict exposure)	approvals (superimposed mixed-mode)
$\left\langle \hat{r}\right\rangle$	0.05661	0.01147	0.4250
normalized $\left\langle \hat{r}\right\rangle$	0.09656	0.01957	0.7250

These patterns may be associated with labyrinthine contagion patterns produced by the generalized SIRS (multi-city) network model for the top-left quadrants of figure 2 across all considered values of ζ. For a high susceptibility acquisition ζ>1, these are configurations where the unaffected but ‘susceptible’ individuals are risk-averse (i.e. avoid locations with elevated criminal incidence), while the affected individuals have negative adaptive responsiveness (i.e. may seek locations with high criminal activity). For ζ≤1, the labyrinthine patterns are mostly aligned with the left side of the cross-diagonal, formed by $\alpha^{S}-\alpha^{I}=50$, where 50 is the maximum range of these parameters. We may assume that such polarizing behaviours produce a segregation of criminogenic environments.

### 2.8. Case study: conflict exposure during COVID-19-related protests in NSW, Australia

We also examined data on conflict exposure during COVID-19-related protests between July and December 2021 in NSW reported by the Armed Conflict Location and Event Data Project (ACLED) [66]. This dataset includes a list of protest events with fields such as administrative area (both state- and LGA-level), a note/report of the event from verified news sources, and ACLED’s ‘conflict exposure’ measure (the estimated number of people living close to a conflict or event) [67]. The note field was filtered by the keyword ‘coronavirus’ to extract only the protests related to COVID-19 restrictions and vaccination mandates, and the state-level administrative area was limited to the state of NSW.

Figure 7 (right column) displays LGAs with a higher relative conflict exposure (normalized by the corresponding LGA population) in darker shades for both the choropleth map (figure 7*b*) and the binarized overlaid grid map (figure 7*d*). The ACS $\langle \hat{r}\rangle =0.01957$ (see table 2) falls within the threshold for spots patterns ($0<\langle \hat{r}\rangle \leq 0.04$), which are observed across two quadrants (see figure 2 for susceptibility acquisition ζ>1): the top left (polarization) and bottom left (spread of social myths). Arguably, the distribution of the estimated populations exposed to these protest events (e.g. conflict exposure) may be associated with the propagation of social myths within a society. In figure 2, the entire leftmost column ($\alpha^{I}=-50$ and all $\alpha^{S}>-30$) exhibits the spots pattern, suggesting that the observed conflict exposure is characterized by a high negative $\alpha^{I}$.

For $\alpha^{I}=-50$, affected individuals (e.g. those believing a social myth, such as anti-vaxxers, COVID-19 deniers, anti-lockdown libertarians) have negative adaptive responsiveness driving them to seek locations with like-minded (affected) individuals. In contrast, the unaffected (‘susceptible’) individuals who do not share these beliefs may have varying risk-aversion tendencies, i.e. those with a negative risk aversion $\alpha^{S}$ may join these protests and become affected by the myth ($-30<\alpha^{S}<0$), contributing to the social myth spreading; and those with positive risk aversion $\alpha^{S}$ reject it ($0<\alpha^{S}<50$) driving polarization. We hypothesize that either of these behaviours (or a combination of both) may have produced the spots pattern (i.e. pockets of social myths) observed during the NSW COVID-19 protests as captured by the conflict exposure data shown in figure 7*d*.

### 2.9. Case study: real estate businesses and residential building approvals in NSW, Australia

In attempting to capture mixed-mode dynamics during a period of socio-economic turbulence (e.g. a heightened real estate activity), we extracted both the annual real estate business data (for 2022) and monthly residential building approval data (for December 2022) in NSW from the Australian Bureau of Statistics (ABS) [68,69]. These datasets contain the annual number of registered real estate businesses and monthly number of residential buildings (dwelling units) approved per LGA for a chosen time period. These two datasets represent different temporal scales of real estate investment activity: building approvals generally indicate short-term shifts in construction demand, whereas registered real estate businesses capture longer term business formation dynamics which depend on business planning and regulatory frameworks. We superimposed these data in order to capture two modes of real estate activity: this less-than-ideal choice is justified by the absence of a single data source representing both scales.

**Figure 8.**
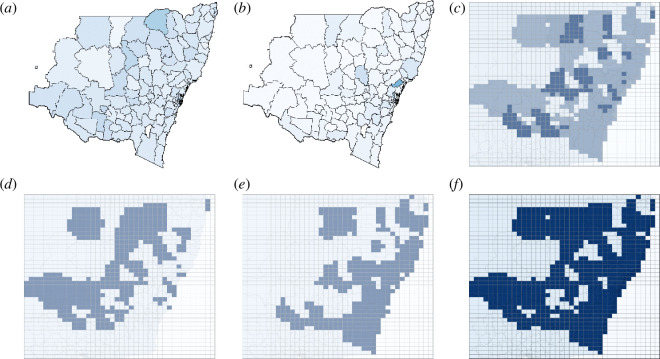
Annual number of registered real estate businesses for 2022 and approved monthly residential buildings for December 2022 are shown per LGA in NSW, Australia. Darker colour highlights LGAs with (*a*) higher numbers of registered businesses in real estate or (*c*) number of residential building approvals, normalized to the LGA’s population. Sub-figures (*b*) and (*d*) show the corresponding redistributed values on the overlaid grid, where areas with higher normalized values (number of businesses or approved residential buildings relative to the LGA’s population) exceeding the mean normalized value across the grid are indicated in blue. These grids are superimposed in sub-figure (*e*), where the darkest areas represent high values for both datasets. Sub-figure (*f*) shows the resultant re-binarized pattern that incorporates both datasets, and can be compared with the mixed-mode pattern produced by unfolding socio-economic turbulence (see figure 3*d*). The normalized ACS $\left\langle \hat{r}\right\rangle$ for this configuration is shown in table 2.

Figure 8 shows LGAs with a higher relative number of registered businesses in real estate (figure 8*a,b*) and residential building approvals (figure 8*c,d*). These resultant patterns are superimposed in figure 8*e*, where the darkest regions are LGAs with high relative values for both datasets. The ACS $\langle \hat{r}\rangle =0.7250$ (see table 2) falls within the threshold for highly connected patterns ($0.4<\langle \hat{r}\rangle \leq 1$). In particular, this highly connected regime is observed in the bottom right quadrant (socio-economic turbulence, comprising asset price bubbles) as a pattern with fine-grained (chequerboard) and course-grained (strands and rings) features. This supports our conjecture that the resultant configurations for the real estate businesses and building approvals are comparable with patterns produced by the dynamics of boom-and-bust investment cycles in our model.

In our model, the chequerboard patterns with overlaid strands and rings are considered mixed mode patterns and are observed below the diagonal on the risk disposition plane across all values of ζ. This highly connected regime (observed in the entire bottom right quadrant) is produced when susceptible individuals have a negative risk aversion (i.e. risk-taking tendency) $\alpha^{S}$ that influences them to take part in the contagion (e.g. engage in investment activities), and affected individuals have a positive adaptive responsiveness $\alpha^{I}$ that drives them to escape the contagion or take profit.

## 3. Discussion

We developed a concise unifying framework connecting elements of risk disposition, such as risk aversion and adaptive responsiveness, to different types of spatial contagion, within a generalized SIRS network model comprising population mobility flows. The maximum entropy approach allowed us to relate system constraints to control parameters, which were interpreted as varying degrees of risk aversion and adaptive responsiveness, thus capturing the bounded risk disposition of affected individuals. One of the parameters quantified the tendency of susceptible individuals to be repelled by risks, ranging from preferring to mix with affected groups (risk taking, i.e. negative risk aversion) to bypassing locations with a higher relative affected population (positive risk aversion). The other parameter quantified the response of the infected individuals to the contagion, ranging from the preference to continue being affected (negative adaptive responsiveness) to fleeing the affected state (positive adaptive responsiveness). Thus, the two considered elements of risk disposition were interpreted as independent dimensions forming two coordinates of the contagion vulnerability space.

Then, we characterized the contagion vulnerability space with respect to the sign of two-bounded risk disposition parameters, with four contagion types emerging as a result: (i) epidemics and natural disasters, (ii) opinion polarization and segregation, (iii) social myth spreading, and (iv) social unrest (socio-economic turbulence). Importantly, the two-dimensional space was extended by an additional aspect—the generalized susceptibility acquisition—which was interpreted for each contagion type as susceptibility to: (i) infection, (ii) prejudice, (iii) believing social myths, and (iv) participating in social disorder.

We explored how the bounded risk disposition and susceptibility shaped pattern formation and phase separation in complex spatial contagions. The simulated patterns were related to Turing patterns observed in various reaction–diffusion and ecological systems [38–40]. In particular, the diffusive predator–prey models with Crowley–Martin type response function [41] or Smith population growth [42] also produced classical Turing patterns. In contrast with these studies, our model of (slow) SIRS-network dynamics, implemented as the Lotka–Volterra predator–prey interactions, was augmented with (fast) risk-driven mobility flows across lattice locations, represented by fraction of affected individuals $\phi^{I}$ and fraction of susceptible individuals $\phi^{S}$. At each iteration of SIRS-network dynamics, the maximum entropy principle (MaxEnt) allowed us to update these mobility flows in a least-biased way, utilizing the perceived location benefits $b_{j}$, dependent on bounded risk disposition parameters $\alpha^{I}$ and $\alpha^{S}$. Thus, the Lotka–Volterra dynamics, complemented by the MaxEnt principle, generated different spatio-temporal patterns in response to distinct risk disposition profiles, and these dynamics were shown to have critical regimes.

Some of the phases exhibited mixed regimes. For example, opinion polarization dynamics showed three distinct patterns (spots, labyrinth and gaps), while epidemic dynamics exhibited gaps, chequerboard and malformed labyrinthine patterns. Most likely, the peculiar irregularities of the latter kind are related to the coexistence of different interacting modes of the dynamic observed in reaction–diffusion systems [70].

Crucially, the identified patterns were shown to be highly sensitive to changes in underlying risk disposition parameters, and exhibited tipping points where even small changes in susceptibility acquisition and perceived local risks and benefits (determined by bounded risk disposition) could lead to drastic and substantial shifts in the resultant patterns emerging at the global level. During spatial contagion, the bounded risk disposition and susceptibility acquisition influence pattern formation in response to potentially conflicting mobility preferences of the susceptible and already-affected individuals. Fundamentally, the critical regimes arise due to the feedback loop between contagion states and population mobility driven by human behaviour [2]. Feedback loops are typically implicated in phase transitions within BLV models [52,71,72], and our study extended this analysis to generic social dynamics.

The identified transitions separate different phases in the contagion vulnerability phase space, suggesting that even minor changes in individual risk disposition can have a profound impact on large-scale group behaviours that arise in real-world contagion scenarios. Importantly, the susceptibility acquisition, generalized as an independent dimension, was shown to exhibit critical regimes across diverse spatial contagion dynamics. In particular, our results suggest that patterns emerging in social SIRS dynamics generally undergo a critical change and become stable when the susceptibility acquisition ζ approaches and exceeds the recovery rate γ.

Several implications arise from the presented findings. Each of the considered contagion types have their profound impact on society. Epidemics and natural disasters severely disrupt human lives and economies [73,74], and examining which spatial patterns may result from interplay of the underlying risk disposition parameters may inform relevant epidemic response and resource distribution policies [75,76].

Understanding the dynamics producing opinion polarization and segregation may also inform societal response to prejudice, preventing or reducing the emergence of stereotypes, discrimination and radicalization—in particular, if the susceptibility (prejudice) acquisition can be mitigated and slowed down over time. This contagion type is quite volatile, with three emergent spatial patterns possible under different combinations of behavioural parameters. Thus, analysis of the parameter combinations may reveal subtle precursors for substantial changes in the population mobility, including short-term migration shifts.

Spreading and formation of social myths, often fuelled by ‘infodemics’, conspiracy theories and misinformation, negatively affect people’s social life and health behaviours. Again, reducing the susceptibility acquisition (the tendency to accept beliefs) via education or awareness campaigns, tailored to predominant behavioural parameters, may refute dangerous propaganda and manipulated narratives or facts. In turn, reinforcing ‘immunity’ to misinformation may reduce personal vulnerability and risks of mental illness, while improving family and social relationships, and access to health care [77].

Complex dynamics mapping to civil unrest contagion were found to involve two disparate scales of interaction, and this indicates that relevant crisis and conflict management policies may need to involve both local and global interventions. This may not only help to identify potential ‘hot-spots’, but also improve social cohesion overall.

While we did not consider stochastic effects, we explored diverse real-world scenarios with different underlying behaviours and dynamics, ranging from pandemic spread and real estate activity, to conflict exposure and criminal records. These scenarios were grounded in fine-grained datasets, capturing real-world stochasticity. Our analysis showed that the identified pattern types remained consistent across these varied sociobiological conditions, with the corresponding percolation order parameters observed within the model-predicted ranges. This consistency suggests that the spatio-temporal dynamics produced by our model are robust against potential stochastic influences inherent in complex social and biological phenomena.

Incorporating stochastic effects, as well as calibration, validation and refinement of specific models based on the developed approach using real-world data remains a subject of future work. Nevertheless, our case studies (geospatial spread of COVID-19; criminal records of assault incidents; conflict exposure during COVID-19-related protests; real estate businesses and residential building approvals) showed promising concordance with the model. We believe that the developed unifying framework will have an impact on understanding not only the specific contagion dynamics, ranging from epidemics to civil unrest, but also their socio-economic and socio-political ramifications and tipping points. The corresponding societal shifts may include new migration patterns, emergence of online communities, as well as unexpected election outcomes, social disparities and economic crises.

Ultimately, the tools supported by the unified spatial contagion framework may guide experts, policy- and decision-makers to proactively model, design and implement contagion-specific targeted interventions, increasing societal resilience in the face of evolving challenges.

## 4. Methods

### 4.1. Canonical SIRS model

The canonical SIRS epidemic model groups individuals into three compartments: susceptible (S), infected (I) and recovered (R) [32], with the compartmental dynamics driven by transmission rate β, recovery rate γ and the loss of immunity ζ. The dynamics of the SIRS model is governed by the following differential equations (see electronic supplementary material):


(4.1) $$\dot{S}=-\frac{\beta }{N}IS+\zeta R,$$

(4.2) $$\dot{I}=\frac{\beta }{N}IS-\gamma I,$$

(4.3) $$\dot{R}=\gamma I-\zeta R.$$

The SIRS model represents a range of immunity dynamics, from short-lived immunity to long-lasting immunity. Notably, it may be reduced to either SIR or SIS forms dependent on the rate of immunity loss [32,34]. When ζ=0, the SIRS model reduces to SIR dynamics. In this case, individuals who recover from the infected state remain immune indefinitely. On the other hand, as ζ→∞, the recovered individuals rapidly lose immunity (with $\frac{1}{\zeta }$ being the duration of immunity), resulting in SIS dynamics (see electronic supplementary material). In the SIS model, recovered individuals become susceptible again immediately upon recovery, without acquiring any permanent or temporary immunity [33,78]. The range of ζ between two extremes (0 and ∞) defines a space of susceptibility acquisition that we explore in this study using the SIRS model.

### 4.2. Generalized SIRS network (multi-city) model of spatial contagion

We consider an L×L lattice of M locations with periodic boundary conditions. Each location i, i∈1,...,M, has a sub-population $P_{i}$, with $I_{i}$ representing affected individuals, $S_{i}$ representing susceptible and $R_{i}=P_{i}-S_{i}-I_{i}$ representing recovered. The set of all $I_{i}$, denoted as I, is the state of the contagion across all M locations. A cost associated with mobility is incorporated into the model, using an adjacency matrix 𝐂, where each element $c_{ij}$ may represent an actual cost of commute, physical distance or any other measure of accessibility. For our purposes, the mobility costs in matrix 𝐂 for the self-interaction ($c_{ii}$) and adjacent neighbour (top, bottom, left and right) mobility are set to 1, while all other $c_{ij}$ elements are set to ∞, limiting mobility from location i to itself and its immediate neighbours.

The generalized SIRS network (multi-city) contagion model (equations 4.1–4.3) is formalized as follows:


(4.4) $$\frac{dS_{i}}{dt}=-\beta \sum_{j,k}\phi_{ij}^{S}(I,C)\phi_{kj}^{I}(I,C)\frac{S_{i}I_{k}}{{\hat{N}}_{j}(I,C)}+\zeta R_{i},$$

(4.5) $$\frac{dI_{i}}{dt}=-\gamma I_{i}+\beta \sum_{j,k}\phi_{ij}^{S}(I,C)\phi_{kj}^{I}(I,C)\frac{S_{i}I_{k}}{{\hat{N}}_{j}(I,C)},$$

(4.6) $$\frac{dR_{i}}{dt}=\gamma I_{i}-\zeta R_{i},$$

where


(4.7) $${\hat{N}}_{j}(I,C)=\sum_{k}S_{k}\phi_{kj}^{S}(I,C)+I_{k}\phi_{kj}^{I}(I,C).$$

The term ${\hat{N}}_{j}(I,C)$ denotes the number of individuals mixing or interacting at location j, whereas γ is the recovery rate, and β is the probability of transmission or infection per interaction. Hence, the second term in equation (4.5) captures the incidence rate of new transmissions from the affected to the susceptible population in location i due to interactions with the affected population in location k, specifically occurring in (mixing) location j at time t. Importantly, mobility flows $\phi_{ij}^{S}$ and $\phi_{ij}^{I}$ are not fixed, but rather change as the contagion unfolds, thus introducing a closed feedback loop between the contagion state and mobility. When ζ→∞, the model (equations (4.4)–(4.7)) reduces to the multi-city SIS model considered by [4].

In addition to the cost $c_{ij}$ associated with mobility from location i to location j, we consider a benefit $b_{j}$ defined by the state of contagion in j as $b_{j}=\frac{P_{j}-I_{j}}{P_{j}}$ (i.e. the fraction of unaffected individuals). At any given time, given the defined cost and mobility benefits, the MaxEnt principle determines flows $\phi_{ij}^{S}$ and $\phi_{ij}^{I}$ by choosing the probability distribution that maximizes Shannon entropy [79,80], while satisfying the following constraints on the mean benefit and the mean cost of mobility for both susceptible and affected individuals:


(4.8) $$B^{I}=\sum_{i,j}I_{i}\phi_{ij}^{I}(I,C)b_{j}/\sum_{i}I_{i},$$

(4.9) $$B^{S}=\sum_{i,j}S_{i}\phi_{ij}^{S}(I,C)b_{j}/\sum_{i}S_{i},$$

and


(4.10) $$C=\sum_{i,j}\left(I_{i}\phi_{ij}^{I}(I,C)+S_{i}\phi_{ij}^{S}(I,C)\right)c_{ij}/\sum_{i}\left(I_{i}+S_{i}\right).$$

We do not consider benefit $B^{R}$ for recovered individuals, because those who recover with immunity do not contribute to the infection spread (i.e. do not affect mixing interactions), while those who recover and lose immunity are considered within the susceptible compartment. The MaxEnt approach yields the least-biased solutions for $\phi_{ij}^{S}$ and $\phi_{ij}^{I}$ [4],


(4.11) $$\phi_{ij}^{S}\left(I,C|\alpha^{S},\omega \right)=Z_{S,i}^{-1}\text{exp}(\alpha^{S}b_{j}-\omega c_{ij}),$$

(4.12) $$\phi_{ij}^{I}\left(I,C|\alpha^{I},\omega \right)=Z_{I,i}^{-1}\text{exp}(\alpha^{I}b_{j}-\omega c_{ij}),$$

where $Z_{S,i}=\sum_{j}\text{exp}(\alpha^{S}b_{j}-\omega c_{ij})$ and $Z_{I,i}=\sum_{j}\text{exp}(\alpha^{I}b_{j}-\omega c_{ij})$. These solutions depend on the Lagrange multipliers $\alpha^{I}$, $\alpha^{S}$ and ω.

The mobility flows are assumed to equilibrate at every time step in response to incremental changes in the system contagion state **I** (i.e. the mobility flows are assumed to have fast dynamics). In contrast, **I** is not assumed to achieve equilibrium instantaneously at every time step, requiring a slower pace over multiple time steps (i.e. the contagion follows slow dynamics).

We initialized each location i on the L×L lattice of locations with a random infected population $I_{i}$ ranging from 0 to 5% of its total sub-population. Integrating equations (4.4)–(4.6) with Δt=0.001 over 10 000 time steps produced distinct resultant spatial patterns dependent on the bounded risk disposition parameters. For SIR dynamics, i.e. ζ=0, no notable patterns are formed beyond insignificant noise values (due to the random initial I values) because all individuals recover.

### 4.3. Critical dynamics

We explored and characterized spatial patterns in the contagion vulnerability space by varying the bounded risk disposition parameters $\alpha^{I}$ and $\alpha^{S}$ across the two-dimensional space while maintaining fixed infection and recovery rates (β = 10 and γ = 5). Additionally, for each combination of $\alpha^{I}$ and $\alpha^{S}$, we examined pattern formation across the space of susceptibility acquisition by varying ζ on a logarithmic scale from 0 (i.e. considering the SIR model) to 1000 (i.e. approaching the SIS model).

Spatial patterns are delineated by statistically characterizing connected clusters within a lattice of locations [4,81]. A cluster comprises neighbouring locations with infection levels exceeding the mean infection value (averaged across all locations), and sites are deemed to belong to the same cluster if they are adjacent laterally or diagonally, with each location having eight immediate neighbours. Once clusters are identified, the system is subjected to a lattice-based percolation analysis, where a key parameter is the probability p(r) of a site belonging to a cluster of size r, normalized as $\hat{r}=\frac{r}{L^{2}}$ [82]. Subsequently, the mean value $\left\langle \hat{r}\right\rangle$ characterizes the resultant spatial pattern on the lattice and allows us to quantitatively distinguish phases and critical regimes. The standard method for computing the average cluster size (ACS) typically excludes the largest cluster. However, our analysis revealed sharper transitions with the inclusion of the largest (percolating) cluster. Additionally, in comparison with the largest cluster size (LCS) used alone as an order parameter, the approach utilizing ACS without excluding LCS successfully delineated different pattern types (regimes).

The colours used in figure 2 are determined by the average size of connected clusters $\left\langle \hat{r}\right\rangle$ (i.e. percolation order parameter, as described in §4.3) using the following resolution-specific thresholds: white when $\left\langle \hat{r}\right\rangle$ is undefined, blue when $0<\langle \hat{r}\rangle \leq 0.04$, purple when $0.04<\langle \hat{r}\rangle \leq 0.4$ and red when $0.4<\langle \hat{r}\rangle \leq 1$. Above the diagonal (see figure 2*a,b*), these thresholds identify the spatial patterns relatively well, distinguishing between spots, labyrinth and gaps. Below the diagonal, all configurations exhibit the chequerboard pattern rather than the gaps pattern, and therefore an additional order parameter, such as staggered magnetization (a sum of the average magnetization per site of all the sub-lattices), is needed [4].

### 4.4. Case studies: data sources and methods

Population data and LGA boundaries were taken from the ABS [83,84]. The LGA-level data for different case studies comprise daily incidence data (number of infected people per LGA) taken from the COVID-19 in Australia dataset (covid19data.com.au) [56]; assault incidence taken from NSW Bureau of Crime Statistics and Research [62]; conflict exposure to COVID-19-related protests taken from ACLED [66]; and annual real estate business data and monthly residential building approval data in NSW taken from the ABS [68,69].

To compare the percolation order parameter $\left\langle \hat{r}\right\rangle$ of the resulting geospatial contagion patterns with those identified by the generalized SIRS network (multi-city) model, we carried out a discretization of the normalized LGA-level data (number of affected people over population). This was done by overlaying a 40×40 grid onto the NSW geographical map and assigning the resulting latitude/longitude cells as bins for redistributing normalized values based on the overlapping LGAs-cell areas [85,86]. A schematic diagram of this data aggregation and discretization process is shown in electronic supplementary material, figure SI.1.

The percolation cluster analysis, described in §4.3, was then applied to the 40×40 grid binarized with respect to the cell-value mean. The resulting percolation order parameter values (ACS of the above-the-mean normalized values), shown in table 1, were also renormalized relative to the maximum ACS observed in a fully percolated (infected) NSW grid. This adjustment was done to discount any influence of the non-reachable space (non-NSW and ocean areas) on the ACSs.

These comparisons are not intended to suggest that we calibrated the generalized SIRS network (multi-city) model to specific contagion processes (e.g. epidemiological characteristics of the Delta and Omicron variants of SARS-CoV-2, assault incidence, conflict exposure, real estate investment). Instead, we aimed to demonstrate, in the spirit of stylized facts [87], that the types of formed contagion patterns and the corresponding ACSs are aligned with those suggested by the model (see §4.3), making it a first step towards further calibration and validation.

## Data Availability

The model source code (for Python 3.9.15, running on a high-performance computing cluster) and grid discretization code are available on Zenodo [88]. Data used for generating the results presented in §§2.3–2.5 of this study are reproducible through an execution of the source code available on Zenodo [88]. Data used for generating the results of the case studies in §§2.6–2.9 can be obtained from various online sources [56,62,66,68,69,83,84] Supplementary material is available online [89].
